# Fecal Metabolomic Signatures in Colorectal Adenoma Patients Are Associated with Gut Microbiota and Early Events of Colorectal Cancer Pathogenesis

**DOI:** 10.1128/mBio.03186-19

**Published:** 2020-02-18

**Authors:** Minsuk Kim, Emily Vogtmann, David A. Ahlquist, Mary E. Devens, John B. Kisiel, William R. Taylor, Bryan A. White, Vanessa L. Hale, Jaeyun Sung, Nicholas Chia, Rashmi Sinha, Jun Chen

**Affiliations:** a Microbiome Program, Center for Individualized Medicine, Mayo Clinic, Rochester, Minnesota, USA; b Division of Surgical Research, Department of Surgery, Mayo Clinic, Rochester, Minnesota, USA; c Metabolic Epidemiology Branch, Division of Cancer Epidemiology and Genetics, National Cancer Institute, National Institutes of Health, Bethesda, Maryland, USA; d Division of Gastroenterology and Hepatology, Mayo Clinic, Rochester, Minnesota, USA; e Carl R. Woese Institute for Genomic Biology, University of Illinois at Urbana-Champaign, Urbana, Illinois, USA; f Department of Animal Sciences, University of Illinois at Urbana-Champaign, Urbana, Illinois, USA; g Department of Veterinary Preventive Medicine, The Ohio State University College of Veterinary Medicine, Columbus, Ohio, USA; h Division of Rheumatology, Department of Medicine, Mayo Clinic, Rochester, Minnesota, USA; i Department of Health Sciences Research, Mayo Clinic, Rochester, Minnesota, USA; University of Michigan Medical School

**Keywords:** carcinogenesis, colorectal adenoma, colorectal cancer, metabolomics, microbiome, microbiota

## Abstract

Colorectal adenomas are precancerous lesions of colorectal cancer (CRC) that offer a means of viewing the events key to early CRC development. A number of studies have investigated the changes and roles of gut microbiota in adenoma and carcinoma development, highlighting its impact on carcinogenesis. However, there has been less of a focus on the gut metabolome, which mediates interactions between the host and gut microbes. Here, we investigated metabolomic profiles of stool samples from patients with advanced adenoma (*n *= 102), matched controls (*n *= 102), and patients with CRC (*n *= 36). We found that several classes of bioactive lipids, including polyunsaturated fatty acids, secondary bile acids, and sphingolipids, were elevated in the adenoma patients compared to the controls. Most such metabolites showed directionally consistent changes in the CRC patients, suggesting that those changes may represent early events of carcinogenesis. We also examined gut microbiome-metabolome associations using gut microbiota profiles in these patients. We found remarkably strong overall associations between the microbiome and metabolome data and catalogued a list of robustly correlated pairs of bacterial taxa and metabolomic features which included signatures of adenoma. Our findings highlight the importance of gut metabolites, and potentially their interplay with gut microbes, in the early events of CRC pathogenesis.

## Abstract (precis)

Colorectal adenomas are precursors of CRC. Recently, the gut microbiota, i.e., the collection of microbes residing in our gut, has been recognized as a key player in CRC development. There have been a number of gut microbiota profiling studies for colorectal adenoma and CRC; however, fewer studies have considered the gut metabolome, which serves as the chemical interface between the host and gut microbiota. Here, we conducted a gut metabolome profiling study of colorectal adenoma and CRC and analyzed the metabolomic profiles together with paired microbiota composition profiles. We found several chemical signatures of colorectal adenoma that were associated with some gut microbes and potentially indicative of future CRC. This study highlights potential early-driver metabolites in CRC pathogenesis and guides further targeted experiments and thus provides an important stepping stone toward developing better CRC prevention strategies.

## INTRODUCTION

Colorectal cancer (CRC) remains the second leading cause of cancer death in the United States (1). Colorectal adenomas, or adenomatous polyps, can progress into malignant tumors by acquiring a series of genetic mutations and are thus considered the major precursor lesions of CRC (2, 3). The transformation process is referred to as the adenoma-carcinoma sequence and is known to be associated with many risk factors, including not only sociodemographic (e.g., age, sex, and race) and medical (e.g., family history) but also lifestyle (e.g., smoking history) and dietary (e.g., high consumption of red and processed meat and low intake of dietary fibers) factors (4, 5). Nowadays, establishing causal mechanistic links between such risk factors and CRC pathogenesis has become highly important for identifying effective primary prevention strategies and for further lowering CRC risk (6).

Recently, the gut microbiota has emerged as a central player mechanistically linking various risk factors to CRC pathogenesis (7–9). Several lines of evidence support the idea that many of the known CRC risk factors are also key determinants of the structure and function of gut microbiota, which in turn influence host metabolism, immune responses, and cancer-driving genomic/epigenomic alterations, thereby affecting CRC development. For example, a higher consumption of red and processed meats, which are high in sulfur-containing amino acids and inorganic sulfur, has been shown to increase abundances of sulfidogenic bacteria such as Bilophila wadsworthia and *Pyramidobacter* spp.; these microbes are known to produce genotoxic hydrogen sulfide in the gut, thereby inducing DNA damage in intestinal epithelial cells and promoting carcinogenesis (10–13). In another example, intake of dietary fibers results in enrichment of *Bifidobacterium* and *Lactobacillus* spp., which are capable of fermenting dietary fibers into short-chain fatty acids (SCFAs) (14). SCFAs are known to exert protective effects against CRC through a variety of mechanisms including modulation of regulatory T cell homeostasis and epigenetic modification in tumor cells via inhibition of histone deacetylase (15, 16). Therefore, it has been posited that reduction in dietary fiber intake, accompanied by changes in gut microbiota composition and SCFA production, is mechanistically linked with increased risk of colorectal adenoma and CRC (17, 18). Given the importance, gut microbiota profiling studies have been extensively conducted using 16S rRNA gene sequencing or shotgun metagenomics techniques to uncover links between the gut microbiota dysbiosis and development of colorectal adenoma and CRC and possibly CRC risk factors (19–24).

However, it is the gut metabolome, rather than the gut microbiota itself, which directly affects CRC development in the above examples (hydrogen sulfide and SCFAs). These examples accentuate the importance of characterizing changes in the gut metabolome along the adenoma-carcinoma sequence to better understand the biochemical consequences of different CRC risk factors and their mechanistic implications in CRC pathogenesis. It is even more desirable to have a paired gut microbiota-metabolome data set to dissect the contribution of gut microbiota in metabolomic profiles, as the gut metabolome is derived not only from microbial metabolism but also jointly from diet and host metabolism. So far, in the context of CRC research, there have been only a few studies which globally profiled both gut microbiome and metabolome simultaneously (25–27). However, all such studies included only CRC patients and controls and lacked patients with adenoma in the study populations. Therefore, it is still not very clear how and which microbes and metabolites interactively trigger or support the early events of CRC development.

In this study, we profiled the fecal metabolome, serving as a proxy of the gut metabolome as it largely reflects gut physiology (28), of patients with adenoma (*n *= 102) and matched controls (*n *= 102) to characterize biochemical signatures associated with the early events of CRC pathogenesis. To the best of our knowledge, this study represents the largest number of patients with adenoma with fecal metabolomics data, more than tripling the current record (29). Moreover, for each sample characterized herein, a paired gut microbiome profile was available as part of a data set that we reported previously (20), providing us a means to decipher the potential interplay between gut microbes and metabolites. Lastly, our study also included patients with CRC (*n *= 36), which enabled us to address questions about signature continuity along the adenoma-carcinoma sequence. In short, we present fecal metabolomic signatures that are characteristics of colorectal adenoma patients and their associations with gut microbiota and CRC pathogenesis. Our unique findings provide a useful resource for guiding further targeted experiments and developing new CRC prevention strategies.

## RESULTS

### Patient groups.

Fecal samples from 102 patients with one or more advanced adenomas (≥1 cm; “adenoma” group) and 102 matched controls without any polyps based on colonoscopy (“control” group; frequency matched to the adenoma group by age, sex, and race) were profiled using the ultraperformance liquid chromatography-tandem mass spectrometry (UPLC-MS/MS) platform at Metabolon, Inc. While the primary aim of this study was to elucidate fecal metabolomic signatures of adenoma in comparison to controls, we additionally profiled fecal samples from 36 CRC patients (“carcinoma” group) in order to gain more insight into the adenoma-carcinoma sequence. The groups did not show any significant differences in terms of potential confounders such as age, sex, race, and history of smoking (Table 1).

**TABLE 1:** Demographics of the control, adenoma and carcinoma groups

Demographic characteristic	No. of patients in group:			*P* value^*a*^
	Control (*n* = 102)	Adenoma (*n* = 102)	Carcinoma (*n* = 36)	
Age (yr)				
50–59	18	17	6	0.994
60–69	49	50	19	
>70	35	35	11	
Sex				
Female	40	40	16	0.833
Male	62	62	20	
Race				
White	95	96	33	0.799
Hispanic	4	2	1	
Black	2	2	2	
Other/unknown	1	2	0	
Smoking history				
Smoker	58	66	18	0.340
Nonsmoker	43	36	18	
Missing	1	0	0	

a Fisher’s exact test was used to calculate the *P* values.

### Metabolomic signatures of adenoma.

**FIG 1:**
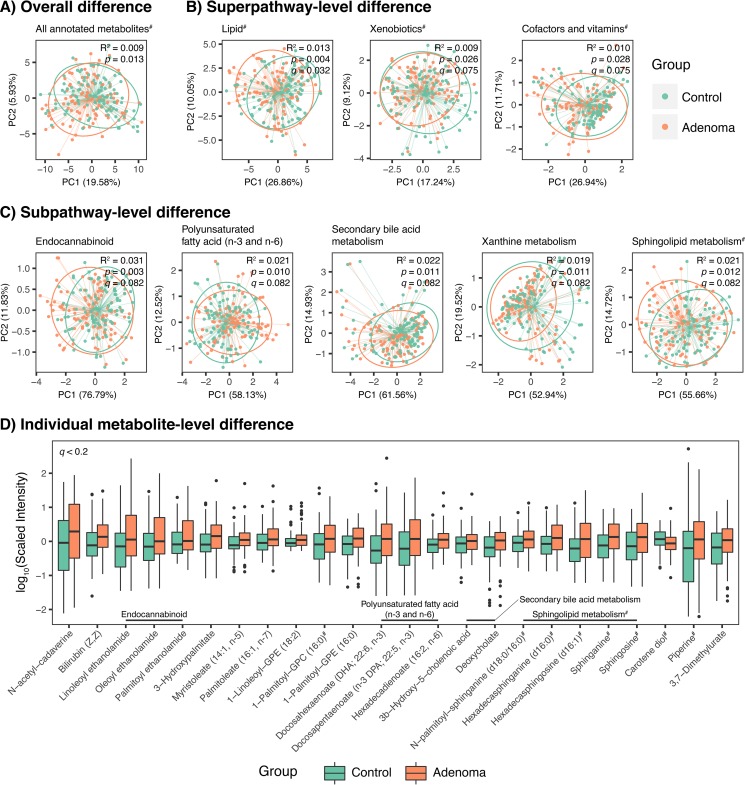
Fecal metabolomic signatures of colorectal adenoma. The adenoma (*n *= 102) and control (*n *= 102) groups were compared in a hierarchical manner. (A) Principal component analysis (PCA) plot based on intensity profiles of all annotated metabolites showing overall difference in metabolomic profiles between the adenoma and control groups. (B and C) Similar PCA plots based on superpathway-level profiles (B) and subpathway-level profiles (C). Metabolon’s definitions of superpathway and subpathway were used. Superpathways and subpathways which displayed distinct pathway-level profiles by patient group tested using PERMANOVA (*q *< 0.1) are shown. In PCA plots, centroids and dispersion of the groups are shown using thin colored lines (from each sample point to corresponding centroid) and ellipses (at 90% confidence level), respectively. All PCA plots are supplemented with PERMANOVA statistics for the group factor. Euclidean distance matrices were used for the PERMANOVA tests. (D) Abundance profiles of differentially abundant metabolites identified by permutation test (*q *< 0.2). #, features also showed differences by sex.

To identify metabolomic signatures of adenoma, we compared metabolomic profiles of patients with adenoma to those of controls in a hierarchical manner using permutation-based statistical tests. First, to assess the overall difference, we performed permutational multivariate analysis of variance (PERMANOVA) on the Euclidean distance matrix between samples based on abundances of all annotated metabolites (Table 2 and Fig. 1A). Factors such as group (adenoma/control), age, sex, race, and history of smoking were tested as predictors of the observed variance between metabolomic profiles using both marginal and adjusted models (Table 2). We found that, regardless of the model of choice, the two factors of group and sex explained small but significant portions of the variance (0.9% and 1.4% of the total variance, respectively; *P < *0.05). This indicates that the fecal metabolomic profiles were, at least in part, dependent on not only presence/absence of adenoma but also sex. Therefore, for our subsequent analyses, we investigated metabolomic differences by adenoma status while adjusting for sex and age, and additionally checked whether the metabolomic features also showed sex differences (Fig. 1; see Materials and Methods).

**TABLE 2:** Factors explaining variance between overall metabolomic profiles of the adenoma and control groups^*a*^

Factor	Marginal model		Adjusted model	
	Variance explained (%)	*P* value	Variance explained (%)	*P* value
Group	0.91	0.013	0.91	0.018
Age	1.19	0.156	1.15	0.191
Sex	1.4	0.004	1.4	0.001
Race	1.54	0.323	1.6	0.244
Smoking history	1.03	0.334	0.87	0.627

a In the first two columns (under “Marginal model”), percent variance explained by a given factor and the corresponding *P* value were derived from a marginal model not adjusted for other factors. In the last two columns (under “Adjusted model”), percent variances explained by factors and the corresponding *P* values were derived from a model adjusted for all factors. PERMANOVA with 999 permutations was used to calculate the *P* values.

To determine groups of metabolites that were associated with the presence of adenoma, we broke down the overall metabolomic profiles into pathway-level profiles using Metabolon’s pathway definition (see Materials and Methods). Using PERMANOVA on Euclidean distance matrices based on the pathway-level profiles, we found that the adenoma group displayed distinct pathway-level profiles in three superpathways (Fig. 1B; “lipid,” “xenobiotics,” and “cofactors and vitamins”) and five subpathways (Fig. 1C; “endocannabinoid,” “polyunsaturated fatty acid” [PUFA], “secondary bile acid metabolism,” “xanthine metabolism,” and “sphingolipid metabolism”) at a false-discovery rate (FDR) of 0.1. All the distinct subpathways, except xanthine metabolism, belonged to the lipid superpathway, suggesting that the overall difference between the groups could be mainly attributable to various classes of lipids and their metabolism. It should be noted that sex differences existed in the pathway-level profiles for all the mentioned superpathways as well as the sphingolipid metabolism subpathway (tested using PERMANOVA).

Next, we sought to identify metabolite-level signatures of adenoma using a permutation test (see Materials and Methods). We identified 24 metabolites that were differentially abundant between the adenoma and control groups at an FDR of 0.2 (Fig. 1D; the relatively larger FDR cutoff was used so as not to miss important metabolites with moderate effects). Interestingly, all the differentially abundant metabolites, except for carotene diol, were found to have increased abundances in the adenoma group compared to the control group. Meanwhile, 19 out of the 24 metabolites belonged to the lipid superpathway. These include three endocannabinoids (more precisely, *N*-acylethanolamines; see Discussion), three PUFAs, two secondary bile acids, and five sphingolipids, again suggesting that lipid metabolites were the main contributors for the overall difference between the metabolomic profiles of the two groups. It should also be mentioned that eight of the 24 differentially abundant metabolites, including all of the five differentially abundant sphingolipids, also showed sex differences in their abundances (tested using the permutation test). In particular, when we further examined sphingolipid levels by simultaneously considering both group and sex factors, female controls displayed strikingly low levels of sphingolipids compared to other metagroups (see Fig. S1 in the supplemental material).

10.1128/mBio.03186-19.2 FIG S1 Differences in fecal sphingolipid levels by patient group and sex. Download FIG S1, PDF file, 0.2 MB. Copyright © 2020 Kim et al. 2020 Kim et al. This content is distributed under the terms of the Creative Commons Attribution 4.0 International license.

### Metabolomic signatures along the adenoma-carcinoma sequence.

We next leveraged data from the carcinoma group to check whether the identified metabolomic signatures were retained in the adenoma-carcinoma sequence. In other words, we aimed to test whether the metabolomic signatures of adenoma can be regarded as early markers of carcinogenesis. Due to the much smaller size of the carcinoma group, we focused the analysis on the adenoma-associated metabolomic signatures to reduce the multiple testing burden. At the subpathway level, we used PERMANOVA to test which of the aforementioned five subpathways that were associated with adenoma were different between the carcinoma and control groups. We found that two subpathways, PUFA and sphingolipid metabolism, showed distinct pathway-level profiles in the carcinoma group in comparison to the control group (Benjamini-Hochberg FDR *q *< 0.1). This suggests that PUFAs and sphingolipids are playing certain roles in carcinogenesis throughout the adenoma-carcinoma sequence.

**FIG 2:**
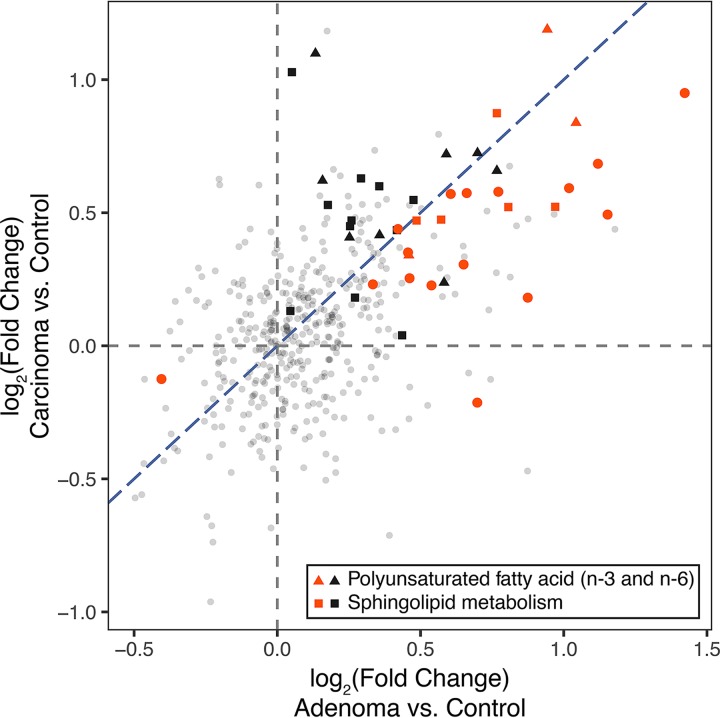
Fold changes in metabolite abundances for the adenoma and carcinoma groups in comparison to the control group. Fold changes for adenoma versus control and carcinoma versus control are shown on *x* and *y* axes, respectively. Metabolites that were differentially abundant in the adenoma group in comparison to the control group are highlighted in red. Triangles and squares regardless of color represent metabolites belonging to two subpathways, “polyunsaturated fatty acid (*n*-3 and *n*-6)” and “sphingolipid metabolism,” respectively. All other metabolites are shown as small gray circles. Blue diagonal dashed line represents the line *y* = *x*.

Next, at the metabolite level, we compared the fold changes in metabolite abundances for the adenoma and carcinoma groups in comparison to the control group (Fig. 2; fold changes for adenoma versus control and for carcinoma versus control on the *x* axis and *y* axis, respectively). Among the 24 metabolites that were differentially abundant in the adenoma group compared to the control group (Fig. 1D), all except for 3-hydroxypalmitate showed directionally consistent changes when the carcinoma and control groups were compared (Fig. 2) (23 of 24 red points were placed in the first and third quadrants; see Fig. S2 for detailed metabolite abundances). However, the majority of the metabolite-level signatures of adenoma seemed to be weakened in carcinoma (Fig. 2) (20 of the red points were located between the *x* axis and the line *y* = *x*). While this may be due to a statistical phenomenon called “winner’s curse,” in which the effect sizes of the largest signals are generally overestimated due to the bias introduced by thresholding (30), this may also indicate that there is no metabolite which clearly shows progressive increase or decrease in its abundance along the disease progression. In addition, this may imply that the metabolite-level signatures are not very robust throughout the adenoma-carcinoma sequence. The pathway-level signatures may serve as more robust markers for the early events of carcinogenesis than the metabolite-level signatures (Fig. 2) (triangles and squares regardless of color indicate PUFAs and sphingolipids, respectively).

10.1128/mBio.03186-19.3 FIG S2 Abundances of differentially abundant metabolites between the adenoma and control groups along the adenoma-carcinoma sequence. Download FIG S2, PDF file, 0.2 MB. Copyright © 2020 Kim et al. 2020 Kim et al. This content is distributed under the terms of the Creative Commons Attribution 4.0 International license.

### Overall association between gut microbiota and metabolome.

In our previous study, moderate but systematic differences in fecal bacterial compositions between patients with and without adenoma (*n *= 233 and *n *= 547, respectively) were identified using 16S rRNA gene sequencing techniques (20). The subjects in the adenoma and control groups of the current study were a subset of the patients included in the previous study. As both fecal bacterial composition and metabolomic profiles were available, and having confirmed that the fecal samples also contained metabolomic signatures of adenoma, we took an integrative, multi-omics approach to uncover potential interplay between gut microbiota and metabolome in the early events of CRC pathogenesis.

**FIG 3:**
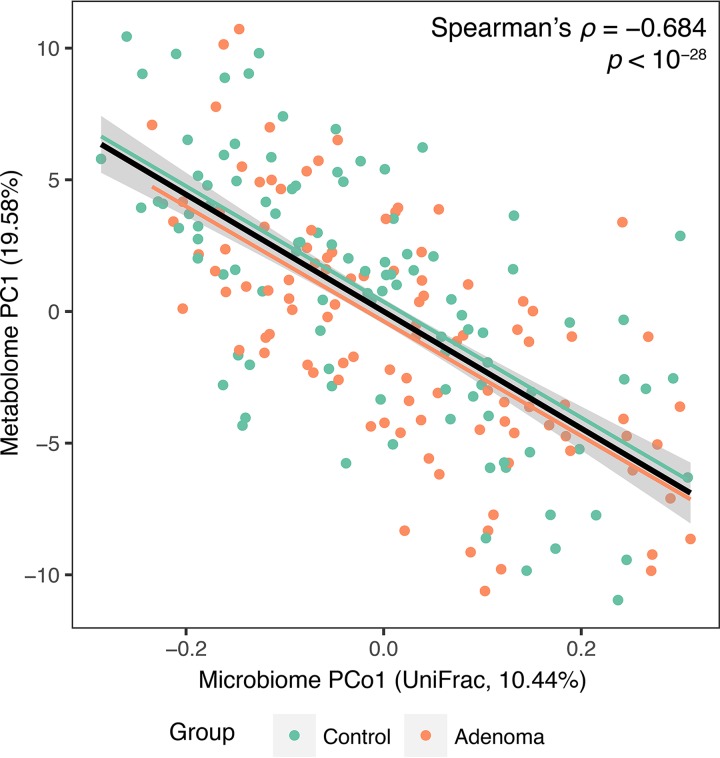
Correlation between the first principal coordinate (PCo1) of microbiome data based on unweighted UniFrac distance and the first principal component (PC1) of metabolomics data. Spearman’s correlation coefficient and its significance were calculated using the adenoma and control samples together (*n *= 204). The black line and gray area show a linear model and its 95% confidence interval describing the overall trend. Green and orange lines represent linear trends for the control and adenoma groups, respectively.

First, to assess the overall association between the bacterial composition and metabolomic profiles, we calculated the correlation between the first principal coordinate (PCo1) of the microbiome data based on unweighted UniFrac distance and the first principal component (PC1) of the metabolomics data. As shown in Fig. 3, we found a very significant correlation between microbial PCo1 and metabolite PC1 using data from the adenoma and control groups (Spearman’s ρ = −0.684, *P < *10^−28^). Correlation coefficients calculated separately for each group were similar to the overall correlation coefficient and showed no significant difference between each other (Spearman’s ρ = −0.686, *P < *10^−14^ for the adenoma group; Spearman’s ρ = −0.673, *P < *10^−14^ for the control group; difference between the two correlation coefficients was tested using Fisher *r*-to-*z* transformation, *P > *0.87). Similar results were obtained when the carcinoma group was considered together with the adenoma and control groups: overall correlation was again very strong (Spearman’s *ρ* = −0.688, *P < *10^−34^), and all groups showed similar correlations (Fig. S3). While the presence of adenoma or carcinoma did not seem to affect the correlation between microbial PCo1 and metabolite PC1, females showed a higher microbial PCo1-metabolite PC1 correlation than males (Spearman’s *ρ* = −0.772, *P < *10^−19^ for females; Spearman’s *ρ* = −0.643, *P < *10^−17^ for males; difference was marginally significant when tested using Fisher *r*-to-*z* transformation, *P < *0.06). Nonetheless, additional 6-group analysis (control/adenoma/carcinoma × sex) showed that there were significant correlations between microbial PCo1 and metabolite PC1 regardless of disease status and sex, even though female groups showed consistently higher correlations than male groups (Fig. S3). Finally, we confirmed that the overall correlation between microbiota and metabolome existed beyond PCo1 and PC1 by using coinertia analysis and Procrustes analysis (Fig. S4), both of which are multivariate statistical methods for testing inter-omics correlations by simultaneously considering multiple principal coordinates or principal components using permutation-based significance assessment methods (31).

10.1128/mBio.03186-19.4 FIG S3 Correlations between microbial PCo1 and metabolite PC1 by patient group and sex. All samples, including samples from the carcinoma group, were used for the calculation (*n *= 240). (A to C) Correlations by group (A), sex (B), and group × sex (C) are shown. (D) Spearman’s correlation coefficients and their significances are provided. Download FIG S3, PDF file, 0.3 MB. Copyright © 2020 Kim et al. 2020 Kim et al. This content is distributed under the terms of the Creative Commons Attribution 4.0 International license.

10.1128/mBio.03186-19.5 FIG S4 Overall association between bacterial composition and metabolomic profiles assessed by coinertia analysis (A) and Procrustes analysis (B). Each arrow represents a pair of microbiome and metabolome data from the same patient. Circles and arrowheads represent microbiome and metabolome data sets, respectively. Both coinertia analysis and Procrustes analysis are based on outputs from ordination analyses (i.e., principal coordinate analysis for the microbiome data set and principal component analysis for the metabolome data set). (A) Coinertia analysis finds coinertia axes that maximize the covariance between the object values of two data sets projected onto the new axes. The first coinertia axis explains most of the covariance, and then the second coinertia axis explains most of the remaining covariance and is orthogonal to the first axis. Coinertia analysis measures the similarity of the object distributions from two data sets along the coinertia axes and reports it as RV coefficient. (B) Procrustes analysis compares the shapes of data sets (the distributions of objects in multidimensional spaces) with corresponding objects. Here, the microbiome data set (circles) is superimposed onto the metabolome data set (arrowheads) and then is moved, rotated, and scaled to best match the shapes (note that the distribution of arrowheads is identical to the distribution of points in Fig. 1A). The similarity of the shapes of data sets is quantified as Procrustes correlation. In both plots, a microbiome data point (circle) is generally placed close to its paired metabolome data point (arrowhead), showing that the bacterial composition profiles are associated with the metabolomic profiles. Meanwhile, arrows are not clearly separated by patient group (color), implying that only a small portion of variance can be explained by the presence of adenoma in both microbiome and metabolome data sets. Download FIG S4, PDF file, 0.3 MB. Copyright © 2020 Kim et al. 2020 Kim et al. This content is distributed under the terms of the Creative Commons Attribution 4.0 International license.

### Correlations underlying the microbiota-metabolome association.

**FIG 4:**
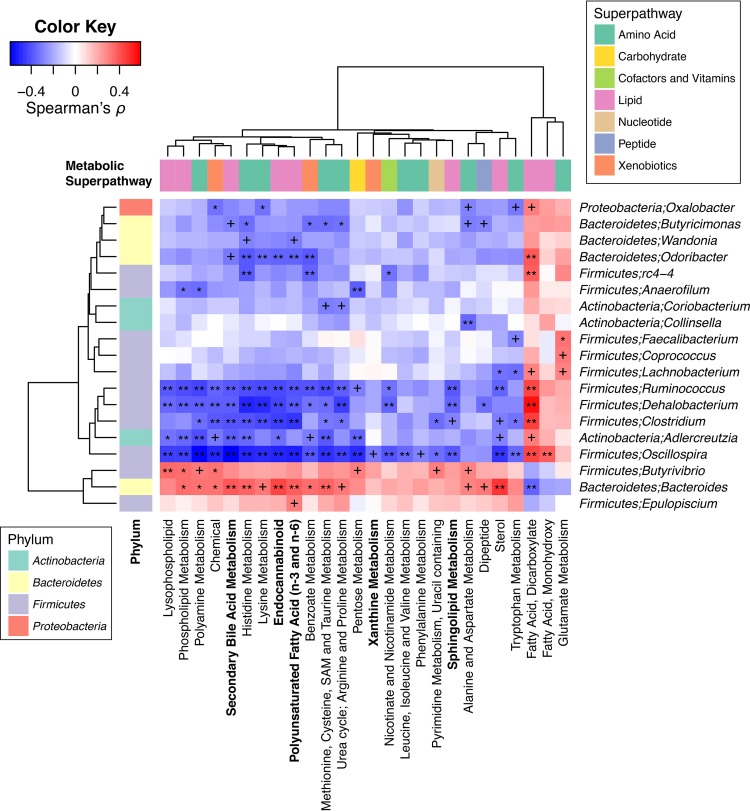
Correlations between bacterial genera and metabolic subpathways. Spearman’s correlation coefficients and their significances were calculated using residual profiles from linear models accounting for the factors, such as patient group, age, sex, race, and history of smoking, to deemphasize associations mainly driven by such factors. The residual profiles were calculated for abundance profiles of each bacterial genus or metabolic subpathway across the adenoma and control groups (*n *= 204). For the subpathway abundance profiles, coordinate values along the first principal components (PC1s) of each subpathway were used. The direction of PC1 was flipped over when a PC1 showed a negative correlation with the averaged intensity profiles of metabolites in the subpathway. Metabolon’s definition of subpathway was used, and only subpathways with at least five metabolites and their PC1s explaining more than 20% of the variance in subpathway-level profiles were considered for the correlation analysis. Features involved in at least one Bonferroni-significant correlation (*q *< 0.05) are shown in the hierarchically clustered heatmap. Names of subpathways which showed distinct pathway-level profiles in the adenoma group compared to the control group are highlighted in bold. +, *q *< 0.1; *, *q *< 0.01; **, *q *< 0.001.

**FIG 5:**
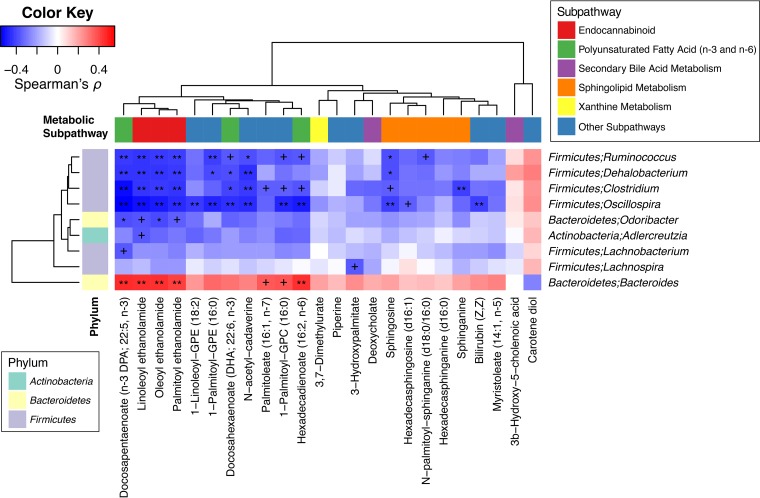
Correlations between bacterial genera and differentially abundant metabolites. Spearman’s correlation coefficients and their significances were calculated using residual profiles from linear models accounting for the factors, such as patient group, age, sex, race, and history of smoking, to deemphasize associations mainly driven by such factors. The residual profiles were calculated for abundance profiles of each bacterial genus or metabolite across the adenoma and control groups (*n *= 204). All annotated metabolites were considered for the calculation, including Bonferroni correction, but only the metabolites that were differentially abundant in the adenoma group in comparison to the control group were shown in the hierarchically clustered heatmap. Bacterial genera correlated with at least one differentially abundant metabolite (*q *< 0.05) are shown in the heatmap. +, *q *< 0.1; *, *q *< 0.01; **, *q *< 0.001.

To investigate which bacterial taxa and metabolite groups (or individual metabolites) were responsible for the overall association between gut microbiota and metabolome, we surveyed individual correlations between genus-level bacterial abundance profiles and subpathway-level (individual metabolite-level) intensity profiles (see Materials and Methods). First, we calculated correlations between 69 genera and 37 subpathways using data from the adenoma and control groups and found several genera that were correlated with many subpathways (Fig. 4). In particular, four genera from the *Firmicutes* phylum (*Clostridium*, *Dehalobacterium*, *Ruminococcus*, and *Oscillospira*) and a genus from the *Actinobacteria* phylum (*Adlercreutzia*) showed Bonferroni-significant negative correlations with 10 or more subpathways. While these genera mainly showed negative correlations with subpathways, they also showed positive correlations with the subpathway “fatty acid, dicarboxylate” (*Oscillospira* was also correlated with the subpathway “fatty acid, monohydroxy”). Meanwhile, *Bacteroides* from the *Bacteroidetes* phylum also displayed Bonferroni-significant correlations with 10 or more subpathways, but in the opposite direction, i.e., it mainly showed positive correlations. Among the subpathways which showed distinct profiles in the adenoma and control groups (Fig. 1C), “endocannabinoid” and “secondary bile acid metabolism” showed significant correlations with all six of the aforementioned genera; “PUFA” was correlated with all but one (not with *Adlercreutzia*); and “sphingolipid metabolism” showed negative correlations with *Dehalobacterium*, *Ruminococcus*, and *Oscillospira*. In contrast, “xanthine metabolism” was only moderately correlated with *Oscillospira*. A similar trend was observed when we investigated individual metabolite-level correlations (Fig. 5).

## DISCUSSION

In this work, we identified metabolomic signatures of colorectal adenoma through untargeted metabolomics profiling of stool samples. To the best of our knowledge, we profiled the largest number of samples from adenoma patients, which enabled us to discover many metabolomic signatures associated with adenoma. Both the pathway- and metabolite-level analyses revealed that the majority of the metabolomic signatures belonged to different classes of bioactive lipids with diverse functions and were associated with CRC development. Although the observed changes in such bioactive lipids were too small to be used as metabolic biomarkers for adenomas (e.g., for diagnostic purposes), these signatures provide key mechanistic insights into early events of CRC pathogenesis (see below). Moreover, exploiting the advantage of having paired microbiome data, we discovered that there existed a strong coupling of the gut microbiota and metabolome and that a part of the metabolomic signatures of adenoma were associated with gut microbes.

Most of the differentially abundant bioactive lipids were elevated in the adenoma group; however, inferring the contribution of such changes to carcinogenesis is not straightforward because the function of a bioactive lipid is dependent on not only its own molecular characteristics but also the balance and relationship with other bioactive lipids (32). For example, we observed that both *n*-3 and *n*-6 PUFAs were elevated in adenoma patients. It is well known that *n*-3 PUFAs have anti-inflammatory and antineoplastic effects (33, 34), and a higher intake of *n*-3 PUFAs lowers the risk of CRC (35, 36). In contrast, *n*-6 PUFAs, which are often working in a competitive way with *n*-3 PUFAs, have proinflammatory effects and may promote carcinogenesis (37, 38). Therefore, the balance between *n*-3 and *n*-6 PUFAs, rather than their respective abundances, may be more relevant to their actual biological activities with regard to carcinogenesis (39, 40). However, this untargeted metabolomics study is limited in that we could not assess the absolute concentrations of metabolites which were required for the calculation of *n*-3:*n*-6 PUFA ratios and for the determination of whether PUFAs are in physiologically relevant concentrations. In another example, we found that sphingosine and other sphingolipids (such as sphinganine and *N*-palmitoyl-sphinganine, which can be converted into sphingosine via ceramide in the host cells) were elevated in the adenoma group. It may seem ironic as these sphingolipids are known to have antiproliferative and proapoptotic effects on various cancer cell lines including human colon cancer (41–43). However, it should be also considered that sphingosine can be readily phosphorylated to form sphingosine-1-phosphate, which exerts proliferative and antiapoptotic effects on cancer cells as opposed to sphingosine (44–46). Moreover, some studies showed that adenomas and cancers have higher expression levels of sphingosine kinase 1, which is responsible for the conversion of sphingosine into sphingosine-1-phosphate, compared to normal mucosa using human colon samples (47–49). Therefore, the balance between sphingosine-1-phosphate and sphingosine (and other sphingolipids) should be assessed to conclusively state the role of sphingolipids in colorectal dysplasia (50, 51). Unfortunately, sphingosine-1-phosphate was out of the coverage of the untargeted metabolomics platform used in this study. Nevertheless, based on the additional observation that PUFAs and sphingolipids were also perturbed in the carcinoma group at the pathway level, we suggest that imbalances in PUFAs and sphingolipids seem to play a significant role in the adenoma-carcinoma sequence and require further investigation, for example, through targeted metabolomics.

Other bioactive lipids elevated in the adenoma group include secondary bile acids and endocannabinoids (*N*-acylethanolamines). Secondary bile acids, including deoxycholic acid, are known to have cytotoxicity on colonic epithelial cells and promote carcinogenesis (52–54). *N*-Acylethanolamines, such as palmitoyl-, oleoyl-, and linoleoyl-ethanolamides (which belonged to the subpathway “endocannabinoid” according to the Metabolon's pathway definition but more precisely are nonendocannabinoids as they are cannabinoid receptor inactive), exert their main biological activities through the activation of peroxisome proliferator-activated receptor α (PPARα) (55–57). However, the role of PPARα in CRC pathogenesis remains controversial (58). Some studies showed that PPARα has anti-inflammatory and antitumor activities in human CRC cells (59). However, another study suggested that PPARα has a tumor-promoting activity in CRC cells through cross talk with the farnesoid X receptor pathway when there exist endogenous bile acids (60). As these two subpathways did not show statistically significant alterations in the carcinoma group in comparison to the control group, bioactive lipids in these classes may be involved in early steps of carcinogenesis rather than later steps.

Through the multi-omics profiling, we were able to investigate the associations between the gut microbiota and metabolome. Regardless of disease status, we observed remarkably strong overall associations between microbial taxonomic and metabolomic profiles using different statistical tests. Such a strong coupling of gut microbiota and metabolome has been reported elsewhere (61, 62). Not only direct interplays between microbes and metabolites, e.g., consumption/production of metabolites by microbes and promotion/suppression of microbial growth by metabolites, but also complex and indirect interplays mediated by other factors, such as diet, host physiology, and immune response, might drive the overall association. Unfortunately, we were not able to quantify the contribution of the latter to the overall association as we did not have measurements for such mediating factors, e.g., dietary fatty acid content (diet), body mass index (host physiology), and fecal calprotectin level (immune response). Nevertheless, it is possible to provide some reasonable explanations for the observed overall associations by surveying the individual correlations between microbial and metabolomic features. For example, PUFAs and endocannabinoids (*N*-acylethanolamines), which were likely to originate from diet (57, 63), are unlikely to be extensively metabolized by gut microbiota; however, they were significantly correlated with multiple bacterial genera. Therefore, this may imply that dietary factors drove a certain part of the gut microbiota-metabolome association in accordance with previous reports which showed that diet can shape both the gut microbiota and metabolome (64, 65). Meanwhile, we also found possibly direct interactions between microbes and metabolites, i.e., positive correlations between *Bacteroides* and secondary bile acids. Some *Bacteroides* species, such as Bacteroides fragilis and Bacteroides thetaiotaomicron, are known to possess bile salt hydrolases and hydroxysteroid dehydrogenases, which are responsible for converting host-produced conjugated primary bile acids into secondary bile acids (66). However, we still want to leave a caution that the links between *Bacteroides* and secondary bile acids may be spurious as there exists a complex cross talk between dietary lipid intake, host metabolism, bile acids, and microbiota (67, 68). Taken all together, our results may indicate that (i) some dietary bioactive lipids (e.g., PUFAs and *N*-acylethanolamines) directly promote carcinogenesis without the involvement of gut microbiota and (ii) nonspecific dietary lipids indirectly contribute to carcinogenesis via relevant changes in host-microbiota cometabolism (e.g., secondary bile acid metabolism).

It is noteworthy that, despite their potential importance in the adenoma-carcinoma sequence, metabolites in the *de novo* sphingolipid biosynthetic pathway, such as sphinganine and *N-*palmitoyl-sphinganine, showed almost no correlations with microbes. While such sphingolipids were likely derived from the host cells rather than microbes or diet, it is well known that some bacterial species, e.g., members of the *Bacteroidetes* phylum, produce sphingolipids that are structurally similar but not identical to mammalian sphingolipids, and such bacterial sphingolipids can modulate the host immune system (69). However, this study is limited in that the metabolomics platform primarily covers human-derived metabolites, not microbiota-derived metabolites. Therefore, in future studies, it would be interesting to focus on bacterial sphingolipids and their roles in the adenoma-carcinoma sequence.

Interestingly, we found some sex differences in our data set. Specifically, we observed that (i) the female control group showed uniquely low levels of sphingolipids compared to other groups and (ii) the overall gut microbiota-metabolome association was stronger in females than males regardless of the presence of adenoma or carcinoma. Nowadays, researchers are becoming increasingly aware of sex differences in CRC as more evidence suggests that risk factors and molecular events leading to the disease are substantially different by sex (70, 71). Here, this work is one of the first studies which highlighted the importance of sex differences in gut metabolome (and potentially its association with gut microbiota) in CRC development. It would be worth investigating whether sex differences in gut metabolome are associated with different paths of CRC development in the future (72, 73). It should be also noted that, in contrast to the fact that we devoted a fair amount of effort to investigating sex differences in our data set, we were not able to study the effect of ethnicity on metabolomic and taxonomic profiles as our study population is predominantly non-Hispanic white. This is reflective of the geographic distribution of ethnic groups in the areas where participating medical centers are located (74). Further studies are warranted to replicate our findings across different ethnicities.

This study is exploratory in nature. It is a limitation of our study that we could not provide clear causalities for our findings. First, the cross-sectional nature of this study does not allow us to determine the time order of variables which is required for establishing causal relationships. Second, we are not able to guarantee that the observed associations are not spurious at all, which is a condition required for the causal inference, in some degree, due to the lack of information on diet and other potential confounding factors. Nevertheless, lists of the metabolomic signatures of adenoma and correlated pairs of microbes and metabolites catalogued in this study would be useful for directing further targeted studies. For example, *in vivo* and *in vitro* experiments using mouse models and cell lines can be designed based on our findings to further investigate cause-effect relationships. Especially, it would be interesting to see if microbes (e.g., *Bacteroides* species) are required for mediating effects of dietary risk factors (e.g., high-fat diets) on malignant transformations via altering bile acid profiles: a study similar in concept already exists in which, however, microbiome has not been considered a variable (75). Drawing a complete interaction map including players like diet, microbiota, and metabolome and highlighting their roles in the adenoma-carcinoma sequence would be a challenging but prominent goal which could lead to better diagnosis and prevention strategies for CRC in future studies.

## MATERIALS AND METHODS

### Study design, subject enrollment, and sample collection.

Individuals were selected from a previous study of 4,482 participants with average risk for CRC (74). Initially, 241 fecal samples were selected from the frozen stool archive for the original study and sent for metabolomics profiling; however, one sample was later excluded from all the statistical analyses as it lacked information on many demographic factors. It should be noted that 780 fecal samples from the original study were previously characterized for microbial composition (20), and a subset of 204 of them (comprising the adenoma and control groups) were selected again for this study. To be more specific, 102 patients with adenoma were first selected to include various types of adenomas in terms of growth pattern (tubular, tubulovillous, villous, or serrated), size, and grade of dysplasia. Then, 102 controls were selected to match the sex, age, and race distribution of the adenoma group. For the carcinoma group, all CRC cases that were available in the original study population were selected (74). More details on the subject enrollment, exclusion criteria, and sample collection processes are available in Text S1 in the supplemental material and the previous studies (20, 74).

10.1128/mBio.03186-19.1 TEXT S1 Supplemental methods used in this study. Download Text S1, DOCX file, 0.02 MB. Copyright © 2020 Kim et al. 2020 Kim et al. This content is distributed under the terms of the Creative Commons Attribution 4.0 International license.

Approval for this study was granted by the Mayo Clinic’s Institutional Review Board. Fecal samples were collected under protocol no. 15-004021, from patients who had previously enrolled under protocol no. 532-00, undergone standard screening colonoscopies, and given consent for the use of their samples in future research studies.

### Microbiota analyses.

Experimental procedures, including DNA extraction from the fecal samples and amplification/sequencing of 16S rRNA genes, were previously described in detail (20). Briefly, DNA was extracted from a core part of the frozen fecal sample, and the V3-V5 region of the 16S rRNA genes was amplified as described previously (76). The sequencing library was prepared at the University of Minnesota Genomics Center, and sequencing was performed using the Illumina MiSeq system at the Mayo Clinic Medical Genome Facility. After sequencing, obtained sequence reads were processed using our custom bioinformatic pipeline called IM-TORNADO with 97% identity threshold for operational taxonomic unit (OTU) assignment (77).

### Metabolomics analyses.

Untargeted metabolomics profiling of the fecal samples through a UPLC-MS/MS platform was performed by Metabolon, Inc. (Durham, NC, USA). Detailed methods are described in Text S1.

### Statistical analyses.

Statistical analyses on metabolomics data were performed using scaled imputed data provided by Metabolon, Inc. Briefly, the raw data were normalized to account for interday difference, which is a result of UPLC-MS/MS run over multiple days, and then the peak intensities were rescaled to set each metabolite’s median equal to 1. Missing values were then imputed with the minimum observed value of the metabolite across all the samples, yielding the scaled imputed data. We further trimmed the metabolomics data to obtain more reliable and interpretable results: we (i) considered only the metabolites detected in at least 80% of the samples from the adenoma and control groups and (ii) discarded metabolites with unknown identities. Abundance profiles for the remaining 462 metabolites were log_10_ transformed and subjected to the following analyses.

To identify metabolomic signatures of adenoma, samples from the adenoma and control groups were considered. PERMANOVA (78, 79) was first performed on the Euclidean distance matrix between samples to assess the effects of factors (group, age, sex, race, and history of smoking) on variance between overall metabolomic profiles (462 metabolites). Both marginal (not adjusted for other factors) and adjusted (adjusted for all the factors) models were tested using the “adonis” function in the R “vegan” package v2.5-2 with 999 permutations. PERMANOVA was also applied on Euclidean distance matrices based on pathway-level metabolite abundance profiles to identify metabolic pathways, where the variance can be explained by the group factor, using models adjusted for sex and age with 999 permutations. For the definition of metabolic pathways, we followed Metabolon’s definition of superpathway and subpathway, and 8 superpathways and 38 subpathways with at least 5 annotated, frequently detected metabolites were considered. To identify differentially abundant metabolites, because some metabolites showed nonnormal distributions, we performed a permutation test based on the *F* statistic of a regular linear model while adjusting for sex and age using an in-house R function. To correct for multiple testing, we performed FDR control using Storey’s *q*-value approach (“qvalue” function in R “qvalue” package v2.10.0) (80). Note that we made another set of tests using models adjusted for the group and age factors to identify metabolomic features that showed sex differences.

To evaluate the adenoma-carcinoma sequence in metabolomics data, we examined whether the metabolomic signatures of adenoma could be consistently found by comparing the carcinoma and control groups. First, for the five distinct subpathways of adenoma, PERMANOVA was performed again using models adjusted for sex and age with 999 permutations to identify subpathways which also showed distinct profiles between the carcinoma and control groups. Then, for each metabolite, we calculated fold changes in mean metabolite abundance for the comparisons adenoma-versus-control and carcinoma-versus-control and checked whether the fold change values for the two comparisons were directionally consistent.

To assess the overall association between bacterial composition and metabolomic profiles, we first reduced the dimensionality of data using ordination techniques, i.e., principal coordinate analysis and principal component analysis for microbiome and metabolome data, respectively (“cmdscale” and “prcomp” functions in R “stats” package). For the principal coordinate analysis of microbiome data, the unweighted UniFrac distance matrix calculated using the OTU table and a phylogenetic tree was used. Then, we calculated correlations between the first principal coordinate of microbiome data and the first principal component (PC1) of metabolome data. We also performed coinertia analysis and Procrustes analysis on the ordinated data using “coinertia” and “RV.rtest” functions in R “ade4” package v.1.7-11 and “procrustes” and “protest” functions in R “vegan” package v2.5-2, respectively.

To identify individual features contributing to the overall microbiome-metabolome association, we computed correlations of bacterial genera with metabolic subpathways and individual metabolites using the data from the adenoma and control groups. As the microbiome data are sparser than the metabolomics data, i.e., have many more zeros, here we applied a looser criterion and considered 69 bacterial genera detected in at least 20%, rather than 80%, of the samples from the adenoma and control groups. We also applied the arcsine square root transformation to relative abundance profiles of bacterial genera to minimize potential bias. For the subpathway abundance profiles, coordinate values along PC1s of each subpathway were used. Again, Metabolon’s definition of subpathway was used, and 37 subpathways with at least 5 metabolites and their PC1s explaining more than 20% of the variance in subpathway-level profiles were considered. Direction of PC1 was flipped over when a PC1 showed a negative correlation with the averaged intensity profiles of metabolites in the subpathway. Then, abundance profiles for the bacterial genera, metabolic subpathways, and individual metabolites were adjusted for group, age, sex, race, and history of smoking, i.e., residual profiles were obtained from linear models accounting for the factors, to deemphasize associations mainly driven by such factors. Using the residual profiles, Spearman’s correlation coefficients and their significances were computed for 2,553 (= 69 × 37) genus-subpathway pairs and 31,878 (= 69 × 462) genus-metabolite pairs. Only Bonferroni-significant correlations (α < 0.05) were presented and discussed in this study.

All the statistical analyses were performed in R version 3.4.4 (R Foundation for Statistical Computing, Vienna, Austria; https://www.R-project.org/).

### Data availability.

The data supporting the findings of this study are available in the supplemental material (Tables S1 and S2). The raw sequence files were deposited in the database of Genotypes and Phenotypes (https://www.ncbi.nlm.nih.gov/gap) with the study accession number phs001204.v1.p1 at the time of original publication (20).

10.1128/mBio.03186-19.6 TABLE S1 Processed metabolomics data used in this study (log_10_ transformed). Download Table S1, XLSX file, 1.5 MB. Copyright © 2020 Kim et al. 2020 Kim et al. This content is distributed under the terms of the Creative Commons Attribution 4.0 International license.

10.1128/mBio.03186-19.7 TABLE S2 Processed bacterial abundance data used in this study (genus-level, arcsine square root transformed). Download Table S2, XLSX file, 0.2 MB. Copyright © 2020 Kim et al. 2020 Kim et al. This content is distributed under the terms of the Creative Commons Attribution 4.0 International license.
