# Supplementary figures and images for: Fecal Metabolomic Signatures in Colorectal Adenoma Patients Are Associated with Gut Microbiota and Early Events of Colorectal Cancer Pathogenesis
Source: mBio. 2020 Feb 18;11(1):e03186-19. doi: 10.1128/mBio.03186-19 (PMC7029137; doi:10.1128/mBio.03186-19)

# **Sphingolipid metabolism**

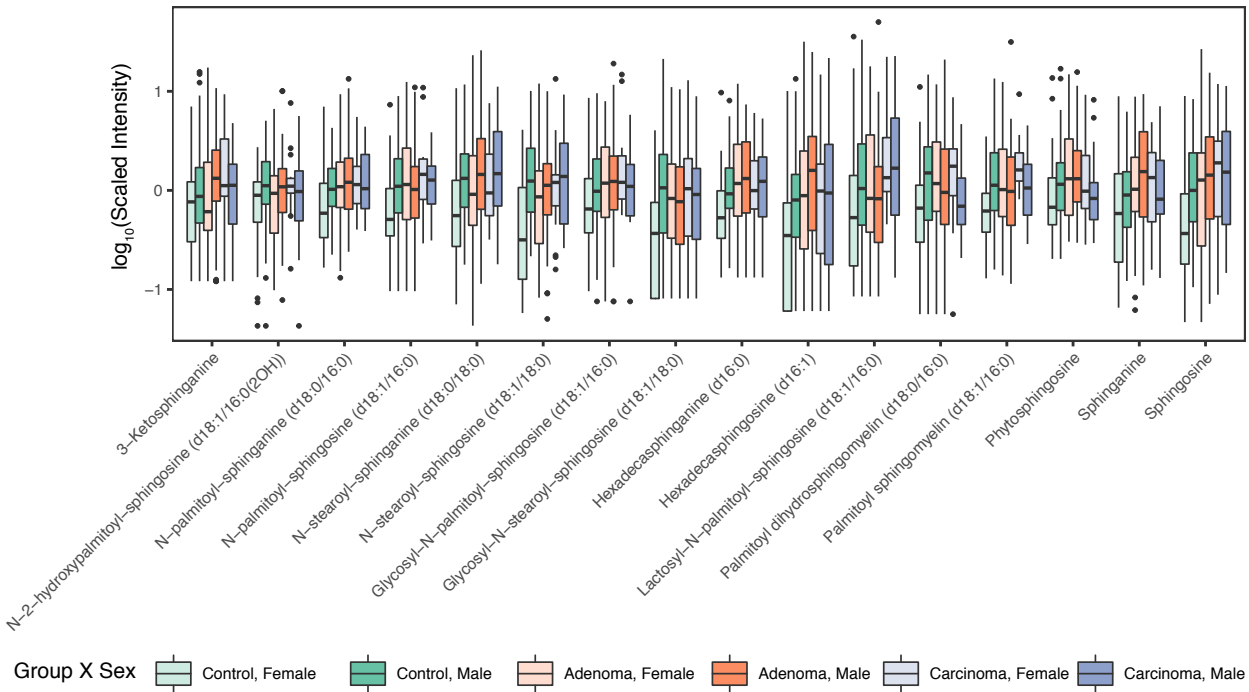

Supplement: FIG S1 [file mBio.03186-19-sf001.pdf]

Directionally consistent (23)

Directionally inconsistent (1)

Monotonic (3)

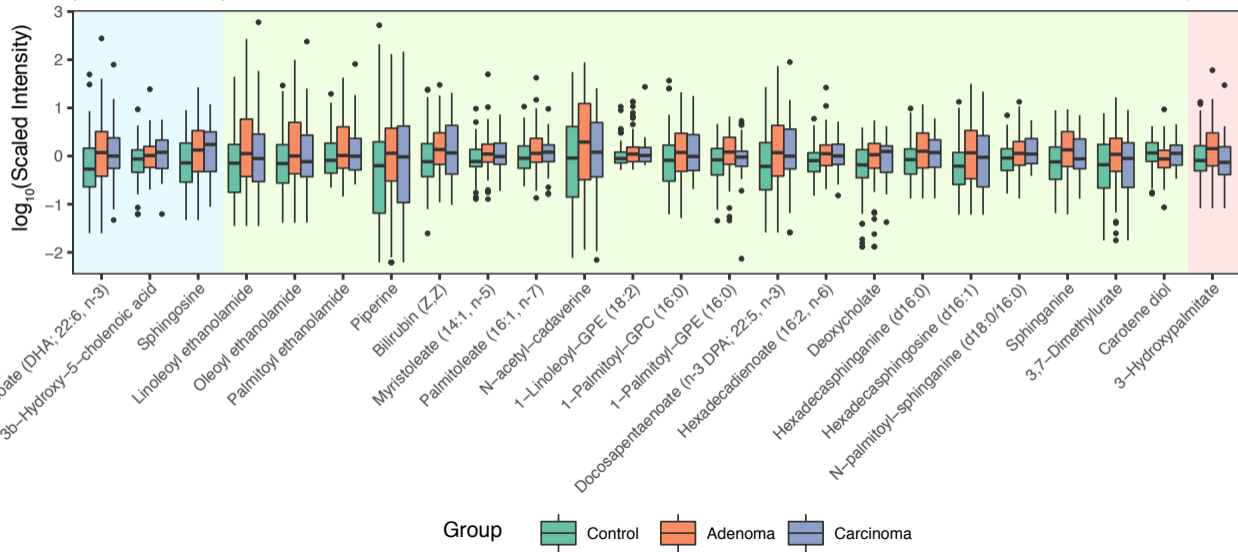

Supplement: FIG S2 [file mBio.03186-19-sf002.pdf]
