# Supplementary material for: Fecal Metabolomic Signatures in Colorectal Adenoma Patients Are Associated with Gut Microbiota and Early Events of Colorectal Cancer Pathogenesis
Source: mBio. 2020 Feb 18;11(1):e03186-19. doi: 10.1128/mBio.03186-19 (PMC7029137; doi:10.1128/mBio.03186-19)
Supplement: FIG S3 [file mBio.03186-19-sf003.pdf]

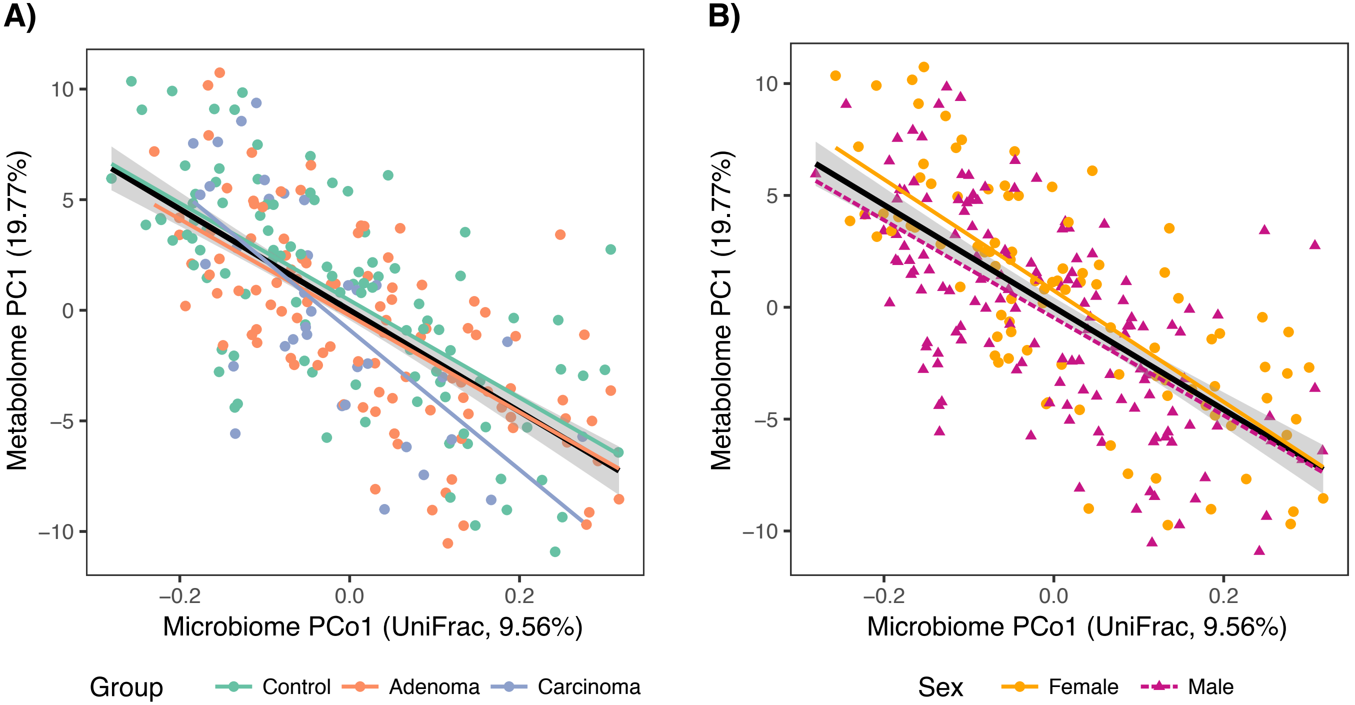

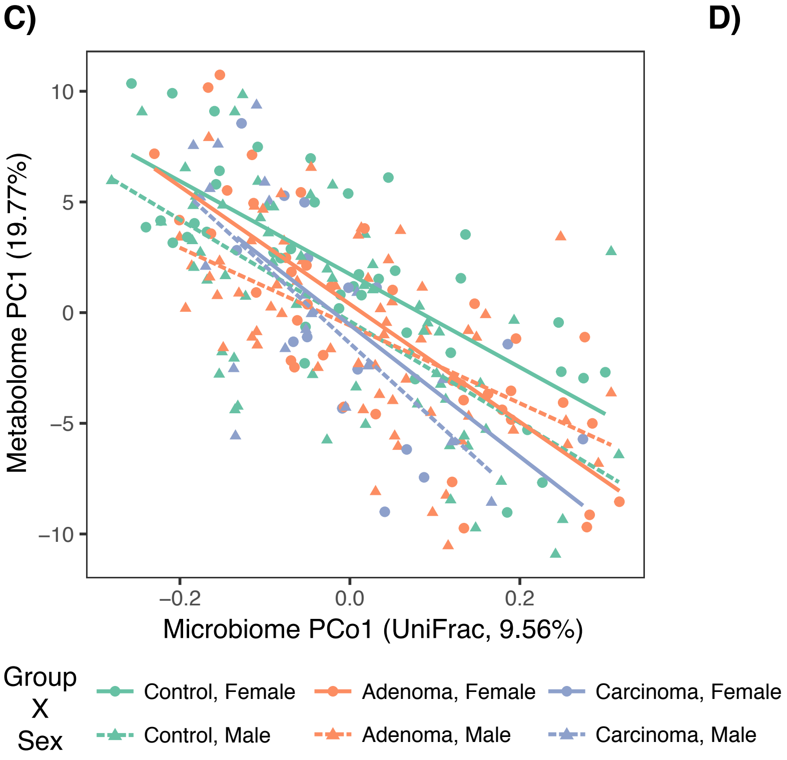

D)

	Female	Male	Overall
**Control**	−0.741 (4.59×10^{-8})	−0.670 (2.50×10^{-9})	−0.684 (2.36×10^{-15})
**Adenoma**	−0.814 (1.65×10^{-10})	−0.576 (9.44×10^{-7})	−0.692 (7.90×10^{-16})
**Carcinoma**	−0.785 (3.13×10^{-4})	−0.681 (9.44×10^{-4})	−0.712 (1.09×10^{-6})
**Overall**	−0.772 (3.63×10^{-20})	−0.643 (3.78×10^{-18})	−0.688 (4.95×10^{-35})

Spearman's ρ
(p-value)
