# Supplementary material for: Fecal Metabolomic Signatures in Colorectal Adenoma Patients Are Associated with Gut Microbiota and Early Events of Colorectal Cancer Pathogenesis
Source: mBio. 2020 Feb 18;11(1):e03186-19. doi: 10.1128/mBio.03186-19 (PMC7029137; doi:10.1128/mBio.03186-19)
Supplement: FIG S4 [file mBio.03186-19-sf004.pdf]

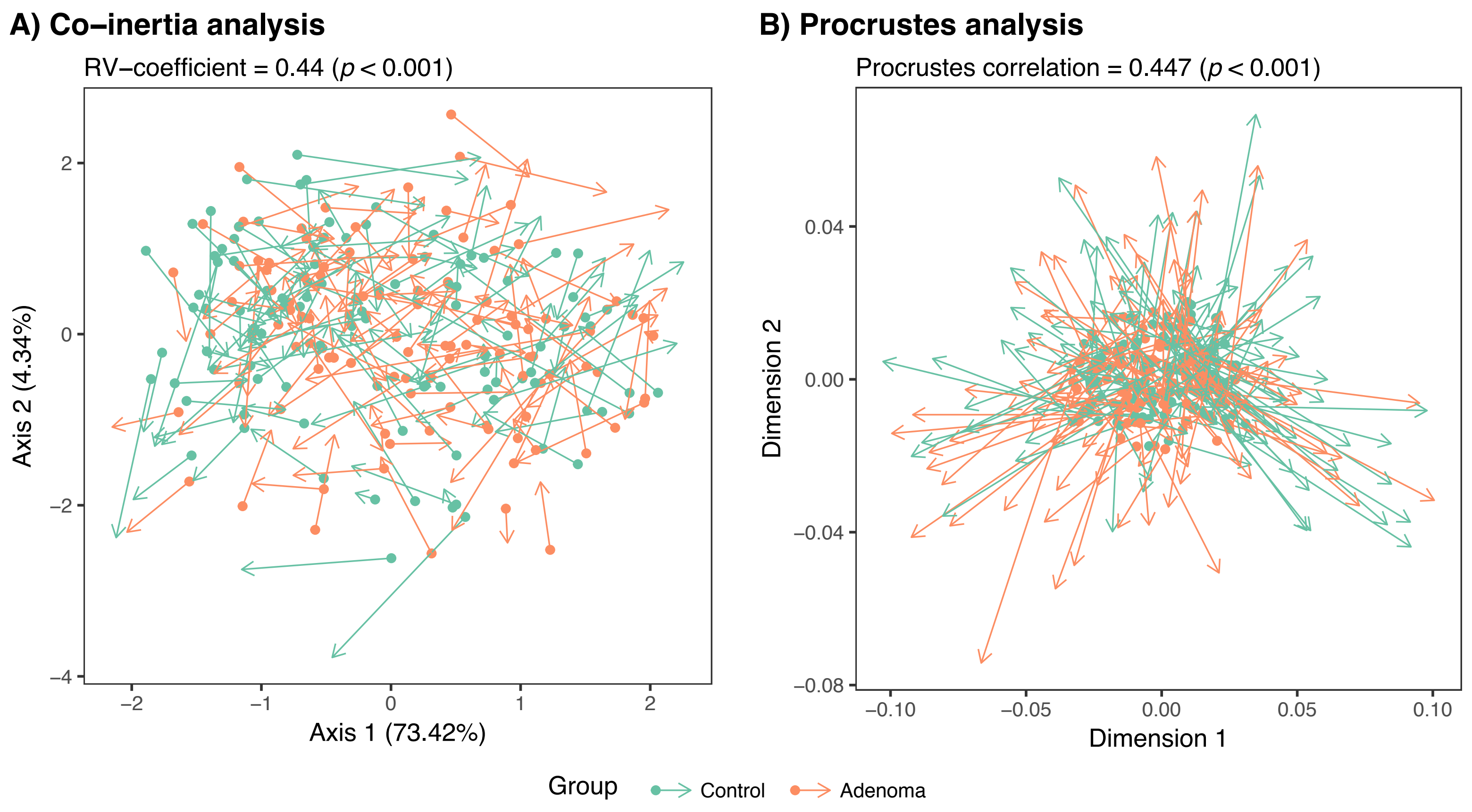
A) Co–inertia analysis
RV–coefficient = 0.44 (p < 0.001)
2
0
−2
−4
−2
−1
0
1
2
Axis 2 (4.34%)
Axis 1 (73.42%)
B) Procrustes analysis
Procrustes correlation = 0.447 (p < 0.001)
0.04
0.00
−0.04
−0.08
−0.10
−0.05
0.00
0.05
0.10
Dimension 2
Dimension 1
Group
Control
Adenoma
