# Supplementary material for: Fecal Metabolomic Signatures in Colorectal Adenoma Patients Are Associated with Gut Microbiota and Early Events of Colorectal Cancer Pathogenesis
Source: mBio. 2020 Feb 18;11(1):e03186-19. doi: 10.1128/mBio.03186-19 (PMC7029137; doi:10.1128/mBio.03186-19)
Supplement: TEXT S1 [file mBio.03186-19-s0001.docx]

**Supplementary Methods**

**Study design, subject enrollment and sample collection**

Individuals were selected from a previous study of 4,482 participants with average risk for CRC (1). Initially, 241 fecal samples were selected from the frozen stool archive for the original study and sent for metabolomics profiling, however, one sample was later excluded from all the statistical analyses as it lacked information on many demographic factors. It should be noted that 780 fecal samples from the original study were previously characterized for microbial composition (2), and a subset of 204 of them (comprising the adenoma and control groups) were selected again for this study. To be more specific, 102 patients with adenoma were first selected to include various types of adenomas in terms of growth pattern (tubular, tubulovillous, villous, or serrated), size, and grade of dysplasia. Then, 102 controls were selected to match the sex, age, and race distribution of the adenoma group. For the carcinoma group, all CRC cases that were available in the original study population were selected (1). All of the fecal samples were collected from asymptomatic patients aged 50 to 80 who presented for a standard screening colonoscopy between 2001 and 2005 at one of the multiple participating medical centers around the United States. Patients self-collected stool samples using a plastic bucket mounted to a toilet seat after the enrollment. Samples were submitted promptly after defecation via express shipping, and were archived at −80 °C as soon as they arrived in a laboratory at the Mayo Clinic, Rochester, MN. All patients received a standard screening colonoscopy after completing the stool collection. Colonic mucosal surfaces were screened for polyps/lesions up to the point of the cecum, and size and location of all polys/lesions were recorded if found. Pathologic examinations on all the polyps/lesions removed from the colon were performed to confirm diagnoses. Our study population was comprised of three groups based on the diagnoses: patients without any polyps/lesions (control group), patients with at least one advanced adenoma (≥1 cm; adenoma group), and patients with colorectal cancer (carcinoma group).

**Metabolomics analyses**

Untargeted metabolomics profiling of the fecal samples through an UPLC-MS/MS platform was performed by Metabolon, Inc. (Durham, NC, USA). Following the receipt by Metabolon, samples were maintained at −80 °C until processed. Several recovery standards were added to the samples before the first step of the extraction process for quality control (QC) purposes. To remove proteins, to dissociate small molecules bound to proteins, and to recover chemically diverse metabolites, proteins were methanol-precipitated under vigorous shaking for 2 mins (Glen Mills GenoGrinder 2000) followed by centrifugation. The resulting extract was divided into five fractions: i) two for two separate reverse phase (RP)/UPLC-MS/MS analyses (optimized for relatively hydrophilic and hydrophobic compounds, respectively) with positive-ion mode electrospray ionization (ESI), ii) one for another RP/UPLC-MS/MS analysis with negative-ion mode ESI, iii) one for HILIC/UPLC-MS/MS analysis with negative-ion mode ESI, and iv) one reserved for backup.

All methods used a Waters ACQUITY UPLC and a Thermo Scientific Q-Exactive high-resolution/accurate mass spectrometer interfaced with a heated electrospray ionization (HESI-II) source and Orbitrap mass analyzer operated at 35,000 mass resolution. The sample extract was dried then reconstituted in solvents compatible to each of the four methods. Each solvent contained a set of standards at fixed concentrations to ensure injection and chromatographic consistency. Two aliquots were analyzed using acidic positive-ion conditions, chromatographically optimized for more hydrophilic and hydrophobic compounds, respectively. In these two methods, the extracts were gradient eluted from a C18 column (Waters UPLC BEH C18-2.1x100 mm, 1.7 µm) using different mobile phases: water and methanol containing 0.05% perfluoropentanoic acid and 0.1% formic acid were used for hydrophilic compounds; and methanol, acetonitrile, water, 0.05% perfluoropentanoic acid, and 0.01% formic acid were used for hydrophobic compounds (and operated at an overall higher organic content). Another aliquot was analyzed using basic negative-ion-optimized conditions using a separate dedicated C18 column. The basic extracts were gradient eluted from the column using methanol and water, however with 6.5mM ammonium bicarbonate at pH 8. The fourth aliquot was analyzed via negative ionization following elution from a HILIC column (Waters UPLC BEH Amide 2.1x150 mm, 1.7 µm) using a gradient consisting of water and acetonitrile with 10mM ammonium formate at pH 10.8. The mass spectrometry (MS) analysis alternated between MS and data-dependent MS^n^ scans using dynamic exclusion. The scan range varied slighted between methods but covered 70-1000 mass-to-charge ratio (m/z).

For the QC, three types of controls were analyzed together with the experimental samples: i) a pooled sample generated by taking a small fraction of each experimental sample (served as a technical replicate throughout the dataset), ii) an extracted water sample (served as process blanks), and iii) a cocktail of QC standards (spiked into every analyzed sample). These allowed instrument performance monitoring and aided chromatographic alignment. Instrument variability was determined by calculating the median relative standard deviation (RSD) for the standards that were added to each sample prior to injection into the mass spectrometers. Overall process variability was determined by calculating the median RSD for all endogenous metabolites (i.e., non-instrument standards) present in 100% of the pooled samples. Experimental samples were randomized across the platform run with QC samples spaced evenly among the injections.

Raw data was extracted, peak-identified and QC processed using Metabolon’s hardware and software. Compounds were identified using a chemical library, which is based on authenticated standards and maintained by Metabolon, that contains the retention time/index (RI), mass-to-charge ratio (m/z), and chromatographic data (including MS/MS spectral data) on all chemicals present in the library. Biochemical identifications are based on three criteria: i) RI within a narrow window of the proposed identification, ii) accurate mass match to the library +/- 10 ppm, and iii) the MS/MS forward and reverse scores between the experimental data and authentic standards. The MS/MS scores are based on a comparison of the ions present in the experimental spectrum to the ions present in the library spectrum. While there may be similarities between these chemicals based on one of these factors, the use of all three data points can be utilized to distinguish and differentiate biochemicals. More than 3300 commercially available purified standard compounds have been acquired and registered for analysis on all platforms for determination of their analytical characteristics. Finally, peaks were quantified using area-under-the-curve.

**Supplementary References**

1. Ahlquist DA, Sargent DJ, Loprinzi CL, Levin TR, Rex DK, Ahnen DJ, Knigge K, Lance MP, Burgart LJ, Hamilton SR, Allison JE, Lawson MJ, Devens ME, Harrington JJ, Hillman SL. 2008. Stool DNA and occult blood testing for screen detection of colorectal neoplasia. Ann Intern Med 149:441-50, W81.
2. Hale VL, Chen J, Johnson S, Harrington SC, Yab TC, Smyrk TC, Nelson H, Boardman LA, Druliner BR, Levin TR, Rex DK, Ahnen DJ, Lance P, Ahlquist DA, Chia N. 2017. Shifts in the Fecal Microbiota Associated with Adenomatous Polyps. Cancer Epidemiol Biomarkers Prev 26:85-94.
